# Application of Telemedicine and Artificial Intelligence in Outpatient Cardiology Care: TeleAI-CVD Study (Design)

**DOI:** 10.3390/diagnostics16010145

**Published:** 2026-01-01

**Authors:** Stefan Toth, Marianna Barbierik Vachalcova, Kamil Barbierik, Adriana Jarolimkova, Pavol Fulop, Mariana Dvoroznakova, Dominik Pella, Tibor Poruban

**Affiliations:** 1 SLOVACRIN & MEDIPARK, Faculty of Medicine, Pavol Jozef Safarik University, Trieda SNP 1, 040 01 Kosice, Slovakia; stefan.toth@upjs.sk (S.T.); kamil.barbierik@siemens.com (K.B.); 2 Cardiology Outpatient Clinic, Kardiocomp s.r.o., Letna 45, 040 11 Kosice, Slovakia; jarolimkova.adriana@gmail.com; 3 School of Medicine, East Slovak Institute of Cardiovascular Diseases, Pavol Jozef Safarik University, Trieda SNP 1, 040 11 Kosice, Slovakia; pfulop@vusch.sk (P.F.); mdvoroznakova@vusch.sk (M.D.); dominik.pella@upjs.sk (D.P.); poruban.tibor@gmail.com (T.P.)

**Keywords:** telemedicine, artificial intelligence, automation, cardiovascular prevention, personalized medicine

## Abstract

**Background/Objectives:** Cardiovascular (CV) diseases remain the leading cause of morbidity and mortality across Europe. Despite substantial progress in prevention, diagnostics, and therapeutics, outpatient cardiology care continues to face systemic challenges, including limited consultation time, workforce constraints, and incomplete clinical information at the point of care. The primary objective of this study is threefold. First, to evaluate whether AI-enhanced telemedicine improves clinical control of hypertension, dyslipidemia, and heart failure compared to standard ambulatory care. Second, to assess the impact on physician workflow efficiency and documentation burden through AI-assisted clinical documentation. Third, to determine patient satisfaction and safety profiles of integrated telemedicine–AI systems. Clinical control will be measured by a composite endpoint of disease-specific targets assessed at the 12-month follow-up visit. **Methods:** The TeleAI-CVD Concept Study aims to evaluate the integration of telemedicine and artificial intelligence (AI) to enhance the efficiency, quality, and individualization of cardiovascular disease management in the ambulatory setting. Within this framework, AI-driven tools will be employed to collect structured clinical histories and current symptomatology from patients prior to outpatient visits using digital questionnaires and conversational interfaces. **Results:** Obtained data, combined with telemonitoring metrics, laboratory parameters, and existing clinical records, will be synthesized to support clinical decision-making. **Conclusions:** This approach is expected to streamline consultations, increase diagnostic accuracy, and enable personalized, data-driven care through continuous evaluation of patient trajectories. The anticipated outcomes of the TeleAI-CVD study include the development of optimized, AI-assisted management protocols for cardiology patients, a reduction in unnecessary in-person visits through effective telemedicine-based follow-up, and accelerated attainment of therapeutic targets. Ultimately, this concept seeks to redefine the paradigm of outpatient cardiovascular care by embedding advanced digital technologies within routine clinical workflows.

## 1. Introduction

Cardiovascular disease management in ambulatory care—encompassing hypertension, dyslipidemia, and heart failure—requires frequent monitoring and timely therapeutic adjustments to optimize outcomes. Conventional in-person follow-up is often sporadic and may fail to detect early clinical deterioration. Telemedicine addresses this through remote patient monitoring and virtual consultations. Current ESC guidelines endorse telemonitoring as integral to heart failure management for reducing hospitalizations and cardiovascular mortality [1].

Contemporary evidence supports telehealth benefits in chronic cardiovascular management. Structured programs with daily HF monitoring demonstrate significant reductions in one-year hospitalization rates and all-cause mortality versus usual care [2]. Large-scale remote management initiatives for hypertension and dyslipidemia yield substantial improvements, with patients receiving remote medication management achieving clinically meaningful reductions in systolic BP (additional 9–10 mmHg) and LDL cholesterol (additional 25–30 mg/dL) at 6–12 months compared to standard interventions [3]. These findings confirm telemonitoring enhances outcomes through early decompensation detection, prompt treatment optimization, and improved adherence.

However, AI integration introduces potential risks requiring systematic evaluation: (1) documentation errors or omissions from AI-generated notes could compromise patient safety; (2) clinician over-reliance on AI summaries may reduce critical review of source data; (3) technical failures could delay time-sensitive clinical decisions; (4) algorithmic bias may affect accuracy across patient subgroups; and (5) implementation barriers including clinician resistance, technical complexity, and workflow disruption may limit real-world adoption. This trial incorporates rigorous safety monitoring, mandatory physician oversight of all AI outputs, systematic error tracking, and comprehensive assessment of implementation challenges [4].

Ambient AI documentation technologies process patient–clinician interactions and autonomously generate draft clinical documentation. A recent RCT demonstrated nearly 50% of clinicians using AI documentation assistants reported reduced after-hours EHR work and decreased documentation frustration, versus 15–20% in controls [5]. In cardiology, AI-assisted tools synthesize complex cardiovascular data into comprehensive structured narratives, with preliminary data suggesting substantial efficiency gains—one cardiologist documented a 1.6 min average chart completion time with over 2 h of daily time savings [6,7].

The novelty of our approach lies in the synergistic integration of three components: (1) continuous telemonitoring generating high-frequency physiologic data, (2) AI-powered automated synthesis and interpretation of this data stream, and (3) AI-assisted clinical documentation reducing physician administrative burden. Unlike prior telemedicine studies that increased clinician workload or AI documentation studies limited to in-person encounters, our model addresses both clinical monitoring intensity and documentation efficiency simultaneously. This represents a paradigm shift from AI as an isolated tool to AI as an integral workflow component enabling scalable, intensive remote care.

Despite growing evidence supporting telemedicine and AI independently, critical knowledge gaps persist: (1) no randomized trials have evaluated integrated AI documentation within telemedicine workflows for cardiovascular care; (2) the dual burden of increased data volume from telemonitoring and documentation requirements remains unaddressed; (3) real-world feasibility and clinician acceptance of AI-assisted documentation in cardiology practice is unknown; and (4) cost-effectiveness of combined telemedicine–AI approaches lacks rigorous evaluation. This trial addresses these gaps by testing a fully integrated system combining continuous remote monitoring with AI-driven data synthesis and documentation support, evaluated through rigorous randomized design with comprehensive clinical, workflow, safety, and economic endpoints.

Implementation barriers to this integrated approach include the following: (1) substantial upfront investment in technology infrastructure and training; (2) patient digital literacy requirements potentially excluding vulnerable populations; (3) regulatory uncertainty regarding AI-generated clinical documentation; (4) liability concerns for AI-assisted clinical decisions; (5) workflow disruption during the implementation period; (6) potential widening of health disparities if technology access is inequitable; and (7) sustainability concerns if ongoing technical support and maintenance requirements are prohibitive. Our trial design explicitly addresses these concerns through structured training protocols, technical support infrastructure, equity-focused recruitment, and comprehensive cost-effectiveness evaluation.

We hypothesize this integrated approach will yield the following: (1) superior clinical control through data-informed, timely adjustments; (2) enhanced patient and physician satisfaction through streamlined communication and reduced administrative burden; (3) maintained or improved safety profiles; and (4) favorable economic outcomes through reduced healthcare utilization and optimized physician productivity. This trial evaluates the feasibility, efficacy, and safety of AI-enhanced telemedicine for managing hypertension, dyslipidemia, and heart failure in ambulatory care versus standard care. Specifically, we aim to (1) determine whether integrated AI–telemedicine achieves superior clinical control of cardiovascular risk factors at 12 months compared to conventional care; (2) quantify the impact on physician documentation time, workflow efficiency, and burnout metrics; and (3) comprehensively assess patient satisfaction, quality of life, and safety outcomes including AI-related documentation errors.

## 2. Artificial Intelligence in Management of Patients

AI is transforming cardiovascular and primary care through enhanced diagnostics, optimized therapeutics, and streamlined workflows, encompassing diagnostic/prognostic analytics, automated documentation, clinical decision support, and remote monitoring.

AI algorithms demonstrate exceptional cardiovascular pathology identification from routine modalities. Machine learning applied to electrocardiography detects left ventricular dysfunction, atrial fibrillation, and ischemic changes potentially missed by conventional interpretation. AI-enabled ECG screening in primary care increased asymptomatic LV dysfunction detection by 32% versus standard assessment, facilitating earlier guideline-directed therapy [8]. Deep learning applied to echocardiography and cardiac MRI autonomously quantifies ventricular function, identifies wall motion abnormalities, and predicts incident heart failure or arrhythmias, enhancing diagnostic precision while reducing interpretation time and democratizing advanced diagnostics [9].

Generative AI ambient scribes capture real-time physician–patient encounters, autonomously generating structured documentation. A multi-center study demonstrated that ambient AI documentation reduced physician burnout from 52% to 39% within one month while improving perceived patient interaction quality, with clinicians reporting decreased after-hours charting and enhanced satisfaction [10]. Contemporary platforms synthesize serial follow-up data, integrate laboratory findings, and identify symptom trends—functioning as dynamic digital assistants preserving clinical continuity [11].

AI-driven decision support analyzes clinical data, cross-referencing patient profiles with practice guidelines and real-world evidence to generate optimized recommendations. AI-generated recommendations achieved diagnostic accuracy comparable to family physicians (55.6% vs. 54.3%) with fewer potentially unsafe therapeutic suggestions [12]. While AI cannot supplant clinical judgment, it standardizes care delivery, reduces practice variation, and minimizes diagnostic/therapeutic omissions. In cardiovascular medicine, algorithms stratify heart failure decompensation risk, guide therapy titration, and identify drug interactions [13].

The most transformative AI applications emerge in telemonitoring for chronic cardiovascular conditions. Machine learning models analyzing continuous physiological data detect subtle deterioration trends. An AI algorithm analyzing multiparameter daily telemetry predicted impending heart failure hospitalizations up to seven days in advance with an AUC of 0.87, substantially outperforming conventional alert systems, enabling preemptive interventions [14]. In hypertension, an AI-guided platform delivering personalized coaching achieved an 8.2 mmHg systolic BP reduction over 24 weeks, nearly doubling the number of patients achieving guideline targets, demonstrating how AI-augmented telemedicine improves outcomes and reduces healthcare utilization [15].

Within ambulatory practice, AI integration into telemonitoring provides proactive disease management. AI tools autonomously collect structured histories, analyze symptom trajectories, and generate concise summaries for physician review. When laboratory data are uploaded, AI interprets temporal changes, flags abnormalities, and suggests therapeutic modifications. Physicians retain decision-making authority while AI enhances efficiency by organizing multimodal data and prioritizing actionable findings [16].

Implementation challenges persist. Interoperability remains fundamental—many applications lack seamless EHR integration, necessitating burdensome parallel workflows [17]. Algorithmic transparency concerns arise from “black box” models providing minimal prediction explanation. Regulatory frameworks struggle to accommodate continuously evolving systems. Ethical considerations persist regarding data privacy, informed consent, and liability attribution.

Future trajectories emphasize collaborative human–AI models wherein AI augments physician expertise—automating routine tasks, synthesizing datasets, and providing real-time decision support while clinicians maintain ultimate accountability. Advancing explainable AI methodologies will build clinical trust. Regulatory agencies are developing frameworks for clinical validation, bias mitigation, and post-deployment monitoring.

**Substantial constraints limit AI adoption in clinical cardiology practice:**

**Technical limitations:** (1) Lack of EHR interoperability requiring manual data transfer; (2) computational requirements for real-time analysis; (3) algorithm degradation when applied to populations differing from training datasets; (4) inability to handle clinical nuance and contextual factors.

**Organizational barriers:** (1) Substantial capital investment without guaranteed return; (2) workflow disruption during implementation; (3) need for ongoing technical support infrastructure; (4) clinician resistance due to training burden and autonomy concerns.

**Regulatory and legal challenges:** (1) Unclear liability framework for AI-assisted decisions; (2) evolving regulatory requirements for clinical AI validation; (3) documentation standards for AI-generated content; (4) informed consent requirements for AI involvement in care.

**Ethical considerations:** (1) Algorithmic bias potentially exacerbating health disparities; (2) data privacy concerns with cloud-based processing; (3) transparency requirements for clinical decision-making; (4) patient preferences regarding AI involvement in their care.

Our trial design explicitly addresses these constraints through comprehensive technical support infrastructure, structured clinician training programs, formal regulatory compliance verification, systematic bias monitoring across patient subgroups, transparent patient consent regarding AI involvement, and rigorous cost-effectiveness evaluation to inform adoption decisions.

Critical risks of AI-generated clinical documentation require acknowledgment: (1) factual errors or hallucinations could propagate throughout medical records, compromising diagnostic accuracy; (2) omission of clinically significant information may delay critical interventions; (3) misinterpretation of ambiguous patient statements could lead to inappropriate treatment; (4) lack of contextual understanding may generate clinically implausible recommendations; (5) automation bias may reduce physician critical evaluation of AI outputs; and (6) medicolegal liability remains unclear when documentation errors originate from AI systems. Mitigation strategies are essential: mandatory physician review and approval of all AI-generated documentation, systematic error tracking with formal classification systems, regular AI performance auditing, clinician training emphasizing critical oversight responsibilities, and explicit documentation of AI assistance in medical records. Our trial incorporates comprehensive safety monitoring specifically targeting AI documentation accuracy.

In conclusion, AI integration represents a paradigm shift toward data-driven, personalized, preventive healthcare. By synergizing AI-enabled history acquisition, automated documentation, decision support, and telemonitoring capabilities, clinicians deliver more efficient, continuous, patient-centered care. Emerging evidence demonstrates improved diagnostic yield, enhanced adherence, reduced administrative burden, and promising cardiovascular risk factor control improvements.

## 3. Telemedicine in Cardiology: Current Evidence and Best Practices

Telemedicine—encompassing synchronous modalities (video/telephone consultations), asynchronous communication, and device-enabled remote monitoring—has evolved to establish a cardiovascular care paradigm, consistently demonstrating risk factor control improvements, earlier clinical intervention, and enhanced patient experience when integrated with structured clinician oversight.

### 3.1. Hypertension: Home Blood Pressure Telemonitoring with Team-Based Management

Home blood pressure telemonitoring (HBPTM) coupled with systematic clinical feedback substantially outperforms usual care and unsupervised self-monitoring. Programs integrating validated automated devices with structured follow-up achieve larger, sustained BP reductions versus application-only interventions [18]. A 2024 systematic review demonstrated greater systolic BP reductions at 6 and 12 months with multicomponent digital interventions—predominantly HBPTM integrated with clinician/nurse case management—versus standard care [19].

Best practice incorporates standardized measurement training, minimum weekly data transmission, protocolized remote medication titration, and proactive outreach. Programs with nurse case management demonstrate superior outcomes; a 2024 RCT showed enhanced BP control with nurse-directed pharmacotherapy titration [20].

### 3.2. Dyslipidemia and Integrated Cardiovascular Risk Management

Telemedicine applications in dyslipidemia emphasize medication adherence reinforcement and timely statin/PCSK9 inhibitor optimization. Remote cardiovascular risk management programs addressing multiple factors produce modest but meaningful metabolic parameter improvements and long-term medication adherence, supporting integrated “tele-prevention” models [21].

### 3.3. Heart Failure: Remote Patient Management and Virtual Ward Models

Heart failure represents the most mature telemedicine domain. The TIM-HF2 RCT demonstrated that multiparameter remote management significantly reduced days lost to unplanned cardiovascular hospitalization or all-cause mortality [22]. A 2024 Cochrane review reports that structured home telemonitoring reduces heart failure hospitalizations, with the greatest effects when threshold-based alerts couple with proactive clinician outreach and remote medication adjustment authority. The 2023 ESC Heart Failure Update incorporated telemonitoring as a Class IIa recommendation for reducing hospitalization risk and improving survival. Post-discharge “virtual ward” models demonstrate reduced unplanned 30-day readmissions and accelerated guideline-directed therapy uptitration [23].

### 3.4. Arrhythmias: Remote Rhythm Surveillance and Diagnostic Access

Remote monitoring of cardiac implantable devices has become standard care. For undiagnosed/paroxysmal arrhythmias, wearable-enabled telemedicine expands detection windows: smartwatch irregularity algorithms demonstrated feasible atrial fibrillation case-finding with high positive predictive value when confirmed by ECG devices [24,25].

### 3.5. Cardiac Telerehabilitation and Secondary Prevention

Cardiac telerehabilitation (CTR) addresses facility-based rehabilitation access barriers. Recent reviews and RCTs demonstrate that CTR achieves functional capacity and risk factor control improvements comparable to traditional rehabilitation, with higher enrollment and completion rates [26].

### 3.6. Program Design Elements Driving Clinical Success

Contemporary evidence identifies critical design elements for successful programs:

1. **Clinician-supervised therapeutic protocols.** The strongest benefits are when telemonitoring couples with action authority—nurse/pharmacist-directed medication titration under protocolized guidelines, scheduled virtual consultations, and explicit physiologic thresholds triggering urgent evaluation [27].
2. **Validated devices and standardized training.** Clinically validated monitors combined with structured patient training and periodic recalibration reduces signal noise and false-positive alerts [28].
3. **Engagement and adherence support infrastructure.** Optimal programs incorporate automated reminders, intuitive interfaces, caregiver access, and multilingual materials; engagement decays without systematic reinforcement.
4. **Equity-centered design principles.** Digital interventions demonstrate disparity-reduction potential when intentionally tailored through device provision, data plan subsidies, and culturally adapted content; without adaptations, programs risk exacerbating inequities.
5. **Data governance and health system integration.** Secure transmission compliant with privacy regulations, role-based clinician access, and bidirectional EHR integration are essential for patient safety and workflow sustainability.

## 4. Integration of AI and Telemedicine: Synergistic Advantages and Implementation Challenges

The convergence of artificial intelligence and telemedicine creates synergistic capabilities transcending the limitations of either technology deployed independently. While telemedicine expands clinical reach and enables continuous monitoring, it simultaneously generates data volumes that overwhelm conventional physician workflows. Conversely, AI algorithms excel at pattern recognition and data synthesis but require large, high-quality datasets for training and validation. The integration of these technologies addresses reciprocal limitations: telemedicine provides the data infrastructure necessary for AI algorithm performance, while AI provides the analytical capacity necessary for telemedicine scalability.

### 4.1. Synergistic Advantages of AI-Enhanced Telemedicine

1. **Enhanced Clinical Surveillance Through Intelligent Monitoring.** Traditional telemonitoring generates alert fatigue through crude threshold-based systems producing high false-positive rates. AI-enhanced systems employ multivariate pattern recognition, analyzing combinations of vital signs, symptoms, and temporal trends to identify clinically meaningful deterioration while filtering benign variations. A retrospective analysis of remote HF monitoring demonstrated that AI algorithms reduced false-positive alerts by 60% while improving sensitivity for detecting clinically significant decompensation from 71% to 89% compared to simple threshold alerts [29]. This intelligent surveillance enables proactive intervention before clinical crises materialize, shifting care paradigm from reactive crisis management to preventive optimization.
2. **Workflow Optimization and Documentation Efficiency.** Telemedicine paradoxically increases the physician documentation burden: virtual consultations require identical charting to in-person visits, while reviewing transmitted monitoring data adds additional documentation requirements. AI-assisted documentation addresses this bottleneck through automated note generation, data summarization, and intelligent pre-charting. A prospective cohort study of AI documentation in 2500 virtual visits demonstrated a 43% reduction in physician documentation time (from 11.2 to 6.4 min per encounter) while maintaining note quality and completeness [30]. This efficiency gain is critical for telemedicine sustainability: without AI assistance, increased monitoring frequency threatens clinician burnout; with AI support, intensive monitoring becomes operationally feasible.
3. **Personalized Clinical Decision Support.** The combination of continuous telemonitoring data and AI-driven analytics enables personalized risk stratification and treatment individualization impossible with episodic in-person assessment. Machine learning models trained on longitudinal patient-specific data can identify individual BP response patterns to medications, predict decompensation risk based on subtle trend changes unique to that patient, and optimize therapy timing based on circadian variation patterns [5]. This represents evolution from a population-based guideline application to genuine precision cardiovascular medicine.
4. **Patient Engagement and Adherence Enhancement.** AI-powered chatbots and conversational interfaces integrated into telemedicine platforms provide 24/7 patient support, answering medication questions, delivering personalized educational content, and providing motivational coaching. A randomized trial of AI chatbot support integrated with BP telemonitoring demonstrated a 28% improvement in monitoring adherence and 31% improvement in medication adherence versus telemonitoring alone [31]. The AI system provided immediate feedback, celebratory messages for goal achievements, and gentle prompts for missed measurements, creating an engagement loop sustaining long-term participation.

### 4.2. Implementation Challenges and Mitigation Strategies

1. **Technical Integration and Interoperability.** Integrating AI algorithms with existing EHR systems and telemonitoring platforms presents substantial technical challenges. Many healthcare institutions employ legacy EHR systems with limited API capabilities, necessitating costly custom integration work. Mitigation strategies include adopting HL7 FHIR standards for data exchange, employing middleware platforms facilitating EHR-agnostic connectivity, and leveraging cloud-based AI services, reducing on-premise computational requirements [32]. Our trial employs REDCap for research data capture interfacing with the STmedical telemedicine platform via standardized APIs, demonstrating feasibility of a modular integration approach.
2. **Clinical Validation and Regulatory Compliance.** AI algorithms require rigorous clinical validation before deployment in patient care, yet regulatory frameworks for AI validation are still evolving. The FDA’s recently released guidance on clinical decision support software establishes a tiered risk classification determining regulatory oversight intensity [33]. Our trial addresses validation requirements through (1) pilot-testing AI algorithms on retrospective datasets before prospective deployment; (2) implementing mandatory physician oversight of all AI outputs; (3) systematic error tracking with pre-defined quality thresholds triggering algorithm retraining; and (4) comprehensive documentation of AI development methodology, training datasets, and performance metrics, enabling regulatory review.
3. **Clinician Training and Change Management.** Successful AI–telemedicine implementation requires substantial clinician training encompassing both technical platform operation and conceptual understanding of AI capabilities and limitations. Physicians must learn when to trust AI recommendations and when to override them based on clinical judgment. Our trial employs a structured training program including didactic sessions on AI principles in cardiology (4 h), hands-on platform training with simulated patient cases (6 h), supervised initial consultations with feedback (first five real patient encounters), and ongoing continuing education addressing emerging challenges. Establishing physician “AI literacy” is a prerequisite for safe and effective implementation [34].
4. **Ethical Considerations and Patient Acceptance.** Patients vary substantially in their comfort with AI involvement in their healthcare. Some view AI as an enhancement improving care quality; others express concerns about algorithmic bias, data privacy, or dehumanization of care. Transparent informed consent is essential, explicitly explaining AI’s role, acknowledging limitations, and emphasizing physician oversight. Our trial consent process includes an educational video demonstrating platform functionality, written materials addressing common concerns, and opt-out provisions allowing participation in telemedicine without AI features (patients would receive standard telehealth without AI documentation assistance). Preliminary survey data from our pilot phase indicated that 78% of patients expressed comfort with AI involvement after educational intervention, compared to 52% before education, highlighting the importance of transparent communication [35].
5. **Cost-Effectiveness and Sustainability.** AI–telemedicine integration requires substantial upfront investment: platform licensing, hardware provision to patients, clinician training, and technical support infrastructure. Long-term sustainability depends on demonstrating favorable cost-effectiveness through reduced hospitalizations, decreased physician overtime, and improved patient outcomes justifying initial expenditure. Economic evaluation is an integral component of our trial, employing societal perspective and capturing costs across the healthcare system: intervention costs (devices, platform, and training), healthcare utilization costs (visits, hospitalizations, and testing), and productivity costs (patient work loss and caregiver burden). Break-even analysis will identify the threshold effect sizes necessary for cost-neutrality, informing future implementation decisions.
6. **Future Directions.** Current systems position AI as a physician-assistive tool requiring human validation of all decisions. Future evolution may enable greater AI autonomy for routine tasks: automatic medication refills when adherence and BP control are stable, automated triage determining the urgency of physician review, and autonomous patient education delivery based on knowledge gaps identified through conversational analysis. However, autonomous AI decision-making introduces novel medicolegal questions regarding liability attribution when adverse outcomes occur. Regulatory frameworks must evolve alongside technological capabilities, balancing innovation enabling improved access and efficiency against safety imperatives, ensuring human oversight of high-stake clinical decisions. Our trial’s comprehensive safety monitoring and error tracking will generate evidence informing these policy discussions. The TeleAI-CVD trial represents the pragmatic integration of AI and telemedicine within contemporary care delivery constraints, prioritizing feasibility and safety while evaluating clinical effectiveness. By comprehensively documenting implementation challenges, workflow impacts, and patient outcomes, this trial will provide an evidence base guiding broader adoption of AI-enhanced telemedicine in cardiovascular care.

## 5. Ethical Framework for AI-Enhanced Cardiovascular Care

The deployment of artificial intelligence in clinical medicine raises profound ethical considerations extending beyond traditional medical ethics principles. While the four pillars of biomedical ethics—autonomy, beneficence, non-maleficence, and justice—remain foundational, AI integration introduces novel ethical dimensions requiring explicit attention: algorithmic transparency and explainability, accountability attribution when AI contributes to adverse outcomes, data governance and privacy in the context of cloud-based AI processing, and equity implications of technology-dependent care delivery.

1. **Algorithmic Transparency and the “Black Box” Problem.** Many contemporary AI systems, particularly deep learning models, function as “black boxes”, producing accurate predictions without providing interpretable reasoning. A cardiologist receives an AI alert, “High risk of HF hospitalization within 7 days (probability: 78%),” but cannot ascertain which specific data elements drove this prediction. This opacity creates ethical tension: physicians are held accountable for clinical decisions but cannot fully evaluate AI recommendations’ validity without understanding their derivation [36]. Explainable AI (XAI) methods attempt to address this through techniques like SHAP (SHapley Additive exPlanations) values quantifying each variable’s contribution to predictions, attention maps highlighting which data elements the model weighted most heavily, and counterfactual explanations demonstrating which changes would alter the prediction [37]. Our trial employs rule-based algorithms for high-stake clinical alerts (explicitly programmed thresholds for BP, weight, and symptoms) ensuring complete transparency, while machine learning models are limited to lower-stake functions (pattern recognition in symptom narratives and trend visualization) where opacity carries less risk. This tiered approach balances AI’s analytical power against transparency requirements.
2. **Informed Consent and Patient Autonomy in AI-Assisted Care.** Traditional informed consent addresses risks and benefits of medical interventions. AI integration requires expanded consent addressing the following: (1) explanation of AI’s role in care delivery—which tasks AI performs and which decisions remain exclusively physician-determined; (2) acknowledgment of AI limitations—potential for errors, inability to account for unique patient circumstances, and the ongoing learning nature of algorithms; (3) data usage disclosure—how patient data trains AI models, whether data is shared with AI vendors, and data retention and deletion policies; (4) alternative options—availability of care without AI involvement and the implications of opting out [38]. Our trial’s consent process employs a layered approach: a brief summary highlighting key points, a comprehensive detailed document for thorough review, an educational video demonstrating platform functionality, and an opportunity for questions with study coordinators. Patients uncomfortable with AI involvement can opt for standard telemedicine without AI documentation features, preserving autonomy while enabling study participation. This approach respects heterogeneous patient preferences regarding technology involvement in their care.
3. **Accountability and Liability in AI-Assisted Clinical Decisions.** When adverse outcomes occur in AI-augmented care, liability attribution becomes complex. If an AI-generated summary omits critical information leading to inappropriate therapeutic decision, is the AI developer liable? The validating physician? The healthcare institution deploying the system? Existing medicolegal frameworks presume human decision-makers bear accountability, but AI’s increasing autonomy challenges this assumption [39]. Our trial design maintains clear accountability: physicians retain ultimate decision-making authority and bear professional responsibility for all clinical judgments. AI functions exclusively as an advisory tool; physicians can and should override AI recommendations when clinical judgment dictates. All AI-generated documentation undergoes mandatory physician review and approval, creating an explicit validation checkpoint. This human-in-the-loop design preserves the traditional accountability framework while enabling AI’s efficiency benefits. Documentation explicitly notes “AI-assisted” to ensure transparency in medical records. Errors attributable to AI are systematically tracked, triggering algorithm refinement and, if severe, a human factor review to determine if the user interface design contributed to misinterpretation.
4. **Data Privacy, Security, and Governance.** AI systems require vast datasets for training and ongoing performance optimization. Patient data transmitted to cloud-based AI platforms raises privacy concerns: Who owns the data? Can it be used for purposes beyond the individual patient’s care? How long is it retained for? Can patients request data deletion? European GDPR establishes the “right to explanation” for automated decisions and “right to erasure,” but practical implementation in healthcare contexts remains challenging given legitimate needs for long-term medical records and AI model training [40]. Our trial implements a comprehensive data governance framework: (1) data minimization—collecting only data necessary for clinical care and research objectives; (2) purpose limitation—data used exclusively for trial purposes, not for unrelated AI model development; (3) encryption at rest and in transit—256-bit AES encryption for stored data and TLS 1.3 for transmission; (4) access controls—role-based permissions limiting data access to authorized personnel; (5) audit trails—logging all data access and modification; (6) data retention policies—anonymized research data retained per regulatory requirements and identifiable clinical data deleted per institutional policies post-trial; (7) patient data access—participants can review their data and request corrections to factual errors.
5. **Algorithmic Bias and Health Equity.** AI algorithms trained predominantly on data from majority populations may perform poorly in underrepresented groups, potentially exacerbating existing health disparities. Studies have demonstrated racial bias in algorithms predicting healthcare utilization, with models systematically underestimating illness severity in Black patients versus White patients with an equivalent objective health status [41]. Similar concerns exist for sex, age, and socioeconomic biases. Mitigation strategies include (1) diverse training datasets—ensuring AI models are trained on representative patient populations; (2) bias testing—systematically evaluating algorithm performance across demographic subgroups; (3) continuous monitoring—tracking outcome disparities during deployment; and (4) transparency—publicly reporting performance metrics stratified by patient characteristics [42]. Our trial employs stratified randomization, ensuring a balanced demographic distribution, with pre-planned subgroup analyses by age, sex, and disease category to detect differential AI performance. If significant disparities emerge, algorithms will undergo retraining with oversampling of underperforming subgroups.
6. **Justice and Equitable Access to AI-Enhanced Care.** Technology-dependent care delivery risks creating a “digital divide” where vulnerable populations lacking smartphones, reliable internet, or technical literacy are excluded from innovative interventions. Paradoxically, these populations often have the highest cardiovascular disease burden and would benefit most from intensive monitoring. Equity-conscious implementation requires proactive measures: device provision programs for patients lacking hardware, subsidized data plans for those unable to afford connectivity, multilingual interfaces accommodating linguistic diversity, simplified interfaces for low health-literacy populations, and alternative options for those preferring traditional care models [43]. Our trial explicitly addresses equity through (1) universal device provision—all intervention patients receive the necessary hardware regardless of their ability to purchase it; (2) connectivity support—mobile hotspots provided for patients lacking home internet; (3) caregiver-assisted protocols—allowing family members to assist with technology operation for patients with physical or cognitive limitations; (4) a multilingual platform—supporting Slovak, English, and Hungarian, reflecting regional linguistic diversity; (5) variable-intensity support—more intensive training and technical support for patients with lower baseline technical literacy. Post-trial, we will evaluate intervention effectiveness across socioeconomic strata to determine whether AI–telemedicine reduces, maintains, or exacerbates disparities.
7. **Professional Integrity and the Human Element in Medicine.** Concerns persist that AI-augmented care may erode physician–patient relationships, replacing empathetic human connection with algorithmic efficiency. Patients may feel their concerns are filtered through AI rather than receiving a physician’s undivided attention. Physicians may become overly reliant on AI recommendations, diminishing clinical reasoning skills through atrophy [44]. A counter-argument emphasizes that by automating routine tasks—data synthesis, documentation, and guideline checking—AI liberates physician time and cognitive resources for genuinely human elements of medicine: empathetic listening, nuanced counseling, and shared decision-making incorporating patient values. Empirical evidence from AI documentation studies demonstrates that physicians using ambient AI scribes report improved eye contact with patients and enhanced perception of physician attentiveness [45]. Rather than replacing human connection, well-designed AI systems may enhance it by reducing distracting administrative burdens. Our trial evaluates this dimension through validated patient satisfaction measures assessing perceived physician empathy, time feeling heard, and satisfaction with communication. We hypothesize that AI-enabled workflow efficiency will enhance rather than diminish relational quality by allowing physicians to focus on patient interaction rather than documentation during consultations.

Ethical AI-enhanced telemedicine requires (1) transparency regarding AI capabilities and limitations; (2) informed consent addressing AI-specific considerations; (3) human oversight preserving physician accountability; (4) robust data governance protecting privacy; (5) proactive bias mitigation ensuring equity; (6) accessible implementation preventing digital exclusion; and (7) ongoing monitoring detecting emerging ethical concerns. The TeleAI-CVD trial operationalizes these principles, generating empirical evidence informing ethical guidelines for AI integration in cardiovascular care.

## 6. Materials and Methods

### 6.1. Study Design

This is a prospective, randomized, controlled, open-label, single-center trial evaluating integration of an AI-based clinical documentation system into telemedicine care delivery for cardiology outpatients. The study will be conducted at the outpatient cardiology clinics affiliated with Kardiocomp Ltd., Košice, Slovakia, enrolling patients with hypertension, dyslipidemia, and/or heart failure meeting predefined eligibility criteria. This protocol adheres to SPIRIT (Standard Protocol Items: Recommendations for Interventional Trials) guidelines for clinical trial reporting and has received institutional review board approval (IRB/ERC of the Kosice Self-Governing Region, Reference number 9483/2025/ODDZ-48993).

**Figure 1:**
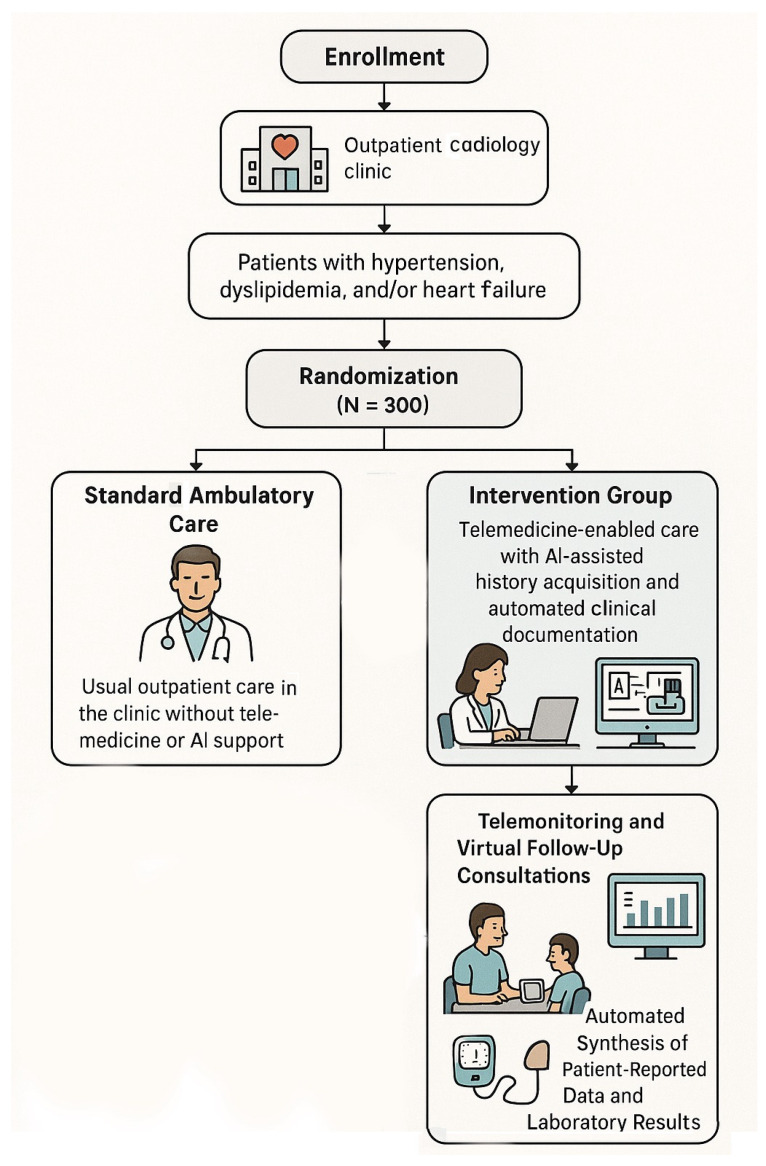
Design of the study.

Approximately 300 patients will undergo 1:1 randomization to (1) the intervention group: telemedicine-enabled care with AI-assisted history acquisition and automated clinical documentation (plus guideline-directed medical therapy) or (2) the control group: standard ambulatory care with conventional clinic visits and traditional documentation, without telemonitoring or AI support (Figure 1, Table 1).

The trial employs an open-label design as the intervention nature precludes the blinding of patients and clinicians to care delivery models. To mitigate bias, outcome data will be collected using standardized protocols, and primary statistical analyses will be performed by a biostatistician blinded to group allocation using coded datasets. This study represents a fully operational protocol with concrete, implementable methods rather than a purely conceptual proposal. All operational details, intervention specifications, data management procedures, and analysis plans are comprehensively defined below to enable immediate implementation upon ethical approval and funding acquisition.

Each participant will be followed for a total duration of 12 months from the date of randomization, with protocol-specified interim assessments conducted at the 6-month timepoint. The trial follows a parallel-group structure with no planned crossover between study arms. The complete study timeline encompasses baseline enrollment and randomization (month 0), an intensive follow-up period with monthly contacts (months 1–3), continued monitoring with bi-monthly contacts (months 4–12), a mid-study assessment (month 6), and a final outcome assessment (month 12).

### 6.2. Patient Population and Eligibility Criteria

**Patient population**: The study will enroll adult patients receiving ambulatory cardiology care for one or more chronic cardiovascular conditions: hypertension, dyslipidemia, and heart failure. The target population comprises patients with suboptimal disease control or those at elevated risk for cardiovascular complications who may derive benefits from intensified monitoring. The trial will focus on a population representative of contemporary cardiology clinic practice:

Hypertension patients: Those with an office blood pressure ≥ 130/80 mmHg despite current therapy or those requiring medication optimization to achieve guideline-recommended targets.

Dyslipidemia patients: Those with LDL-cholesterol above individualized targets based on cardiovascular risk stratification (per ESC/EAS guidelines), requiring initiation or intensification of lipid-lowering therapy.

Heart failure patients: Patients with chronic heart failure (New York Heart Association/NYHA/functional class II–III) who are clinically stable for outpatient management but have documented history of decompensation (hospitalization within the past 12 months) or risk factors for clinical deterioration (e.g., suboptimal medication adherence, comorbidities, and inadequate self-monitoring).

**Inclusion criteria**: Age ≥ 18 years; established diagnosis of hypertension, dyslipidemia, and/or heart failure; access to a smartphone, tablet, or computer at home with reliable internet connectivity for utilization of the telemonitoring application and video consultation platform; capability to measure required physiological parameters at home either independently or with consistent caregiver assistance (e.g., blood pressure and heart rate for hypertension patients; body weight for heart failure patients); willingness and ability to provide informed consent; anticipated availability for the 12-month follow-up period.

**Exclusion criteria**: Unstable or severe acute cardiovascular disease (acute decompensated heart failure, recent myocardial infarction (<4 weeks), uncontrolled cardiac arrhythmia, or other conditions requiring intensive in-person clinical management); NYHA I and NYHA IV heart failure or advanced cardiovascular disease likely to require urgent specialized interventions (such patients may not be appropriate candidates for predominantly outpatient or remote management); cognitive impairment or severe sensory deficits preventing informed consent or technology use; absence of internet-capable device access or discomfort with technology utilization without available caregiver assistance; current participation in another interventional clinical trial that could introduce confounding or conflict with the interventions or outcome assessments of this study; life expectancy < 12 months due to non-cardiovascular disease; pregnancy or planned pregnancy during the study period.

### 6.3. Randomization and Blinding Procedures

Following informed consent, patients undergo 1:1 randomization using computer-generated random sequences with permuted block sizes (blocks of 4 and 6), managed by a biostatistician via a secure web-based randomization system (REDCap). Randomization will be stratified by primary diagnosis (hypertension, dyslipidemia, and heart failure) to ensure balanced distribution of disease categories across study arms.

Due to the intervention’s nature, participant and clinician blinding is infeasible (open-label design). Bias mitigation strategies include the following:

1. Objective outcome measures—blood pressure measurements using validated automated devices and laboratory parameters analyzed by a blinded central laboratory;
2. Identical clinical personnel across arms—delivering guideline-based care;
3. Blinded adjudication of clinical endpoints—hospitalizations and major adverse cardiovascular events) by independent reviewers using standardized criteria;
4. Blinded statistical analysis—using coded identifiers, with unmasking only after primary analysis completion;
5. Standardized data collection protocols—with structured case report forms to minimize measurement bias;
6. Independent data monitoring—by personnel not involved in patient care.

### 6.4. Processing and Availability of Data

All intervention patients receive standardized, clinically validated monitoring equipment:

Blood Pressure Monitoring:

- Device: Omron Evolv (model BP7000) or equivalent validated oscillometric device.
- Validation: Meets ESH International Protocol and AAMI standards.
- Calibration: Annual calibration verification; replaced if accuracy drift detected.
- Transmission: Automatic Bluetooth pairing with STmedical mobile application.
- Training: In-person demonstration with return demonstration; written instructions provided.
- Measurement protocol: Seated position, 5 min rest, arm supported at heart level, 2 measurements 1 min apart.

Weight Monitoring (Heart Failure Patients):

- Device: Withings Body+ or equivalent with 0.1 kg precision.
- Validation: Verified against calibrated clinic scale at baseline.
- Transmission: Wi-Fi-enabled automatic data upload.
- Measurement protocol: Daily morning measurement, same time, after voiding, minimal clothing.

Point-of-Care Laboratory Testing:

- Lipid panels: Venipuncture at certified laboratories with results uploaded to the platform via secure HL7 integration.
- NT-proBNP (HF patients): Obtained at protocol timepoints (baseline, 6, and 12 months).
- Electrolytes and renal function: Standard laboratory testing per protocol.

**Figure 2:**
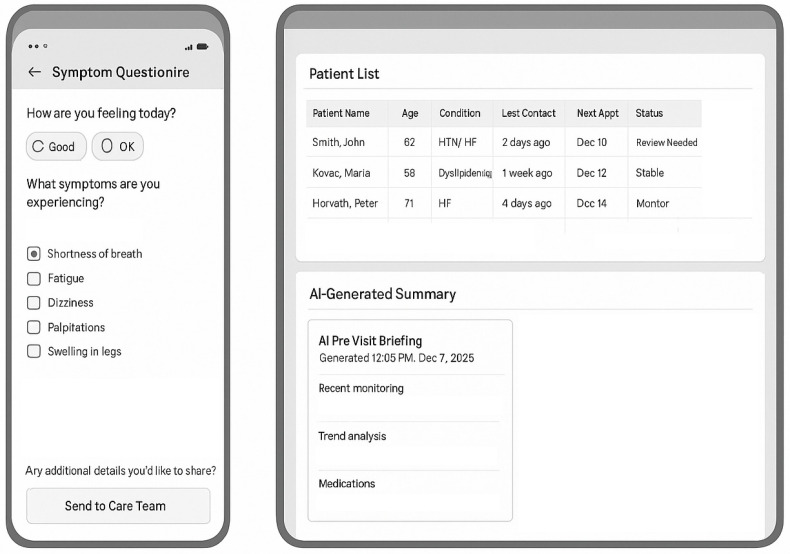
STmedical platform user interface: patient and physician dashboards (draft).

Mobile Application (STmedical Platform) (Figure 2):

- Compatibility: iOS 13+ and Android 9+; tablets provided to patients without compatible devices.
- Features:
  ▪ Real-time vital sign display with trend graphs.
  ▪ Symptom questionnaire interface.
  ▪ Medication tracker with reminders.
  ▪ Secure messaging with care team.
  ▪ Video consultation interface (HIPAA-compliant, 256-bit encryption).
  ▪ Educational content library.
  ▪ Technical Support: 24/7 helpline; average response time < 2 h; in-person troubleshooting available.

Internet Connectivity Support:

- Patients lacking adequate home internet receive mobile hotspot devices (4G LTE).
- Data costs covered by study budget.
- Backup plan: telephone-based data reporting for technical failures.

Quality Control Measures:

- Monthly automated device functionality checks.
- Quarterly patient-reported device performance surveys.
- Replacement devices provided within 24 h for malfunctions.
- Data transmission failures trigger automatic alerts to study coordinators.

**Structural Virtual Follow-Up Consultation Protocol**

Pre-Consultation Preparation (48 h before appointment):

1. Automated Patient Reminders: SMS and app notifications with appointment details.
2. Pre-Visit Questionnaire Deployment:
  (a) Symptom Assessment Module
    - Chest pain/discomfort (character, frequency, triggers, and duration).
    - Dyspnea assessment (NYHA class screening questions and exertional limitations).
    - Palpitations, syncope, and presyncope episodes.
    - Lower extremity edema (visual analog scale + photo upload option).
    - Fatigue/functional capacity changes.
  (b) Medication Adherence Module:
    - Morisky-8 scale automated scoring.
    - Missed dose quantification.
    - Side effect reporting (structured checklist).
    - Medication access barriers.
  (c) Disease-Specific Modules:
    - Hypertension: Headaches, visual changes, and recent BP readings.
    - Heart Failure: Weight trajectory, orthopnea, paroxysmal nocturnal dyspnea, and exercise tolerance.
    - Dyslipidemia: Muscle symptoms (statin-related myalgia screening).
  (d) Lifestyle Factors: Diet adherence, physical activity minutes/week, smoking status, and alcohol intake.
3. AI-generated pre-visit summary (created 24 h before consultation):
  - Synthesizes telemonitoring data from the preceding 2 weeks.
  - Flags abnormal trends (BP elevations, weight gain, and declining data transmission).
  - Compares current vs. previous visit parameters.
  - Integrates recent laboratory results.
  - Highlights patient-reported concerns requiring discussion.
  - Generates a preliminary assessment and suggested discussion topics.

Virtual Consultation Structure (20–30 min duration):

Phase 1: Rapport and Technical Check (2–3 min)
  - Video/audio quality verification.
  - Privacy confirmation (patient in confidential space).
  - Agenda setting.
Phase 2: Symptom Review (5–7 min)
  - Physician reviews AI-generated symptom summary.
  - Clarifies concerning responses from questionnaire.
  - Conducts focused history relevant to patient-reported changes.
  - Visual assessment when appropriate (edema and respiratory effort).
Phase 3: Data Review and Interpretation (8–10 min)
  - Review telemonitoring trends displayed on shared screen:
    ▪ BP trend graphs with target zone overlay.
    ▪ Weight trajectory (HF patients).
    ▪ Measurement frequency and timing patterns.
  - Laboratory result interpretation:
    ▪ Comparison to previous values and guideline targets.
    ▪ Discussion of clinical significance.
  - AI-generated interpretation presented with physician commentary
Phase 4: Therapeutic Decision-Making (5–8 min)
  - Assessment of current therapy effectiveness.
  - Medication adjustments when indicated:
    ▪ Hypertension: Dose titration or additional agent per JNC-8/ESC guidelines.
    ▪ Heart Failure: GDMT optimization per ESC guidelines.
    ▪ Dyslipidemia: Statin intensity adjustment, ezetimibe/PCSK9i consideration.
  - Prescriptions transmitted electronically.
  - Rationale explanation and shared decision-making.
Phase 5: Patient Education and Action Plan (3–5 min)
  - Reinforcement of monitoring protocol.
  - Lifestyle modification counseling.
  - Symptom red flags requiring urgent contact.
  - Next appointment scheduling.
  - Opportunity for patient questions.
Phase 6: Documentation (Physician workflow)
  - Review of AI-generated encounter note draft.
  - Physician edits, additions, and approval.
  - Finalization in HER.
  - Patient receives visit summary via app within 2 h.
Standardized Clinical Decision Support Algorithms Embedded in Consultation:
Hypertension Management Algorithm:
  - If average home BP ≥ 135/85 mmHg over 2 weeks:
    ▪ Stage 1: Medication adherence review → lifestyle counseling intensification → consider dose increase.
    ▪ Stage 2: Add second agent from complementary class per ESC algorithm.
    ▪ Stage 3: Specialist referral for resistant hypertension.
Heart Failure Management Algorithm:
  - If weight increase >2 kg in 3 days or worsening symptoms:
    ▪ Assess volume status markers (orthopnea, edema, and BP trends).
    ▪ Diuretic adjustment (dose increase or additional agent).
    ▪ Consider urgent in-person visit or telehealth-guided clinic visit.
    ▪ NT-proBNP measurement if not recently obtained.
  - GDMT optimization pathway:
    ▪ Systematic review of ACEi/ARB/ARNI, beta-blocker, and MRA status.
    ▪ Dose titration schedule per ESC recommendations.
    ▪ SGLT2i initiation if not contraindicated.
Dyslipidemia Management Algorithm:
  - Risk stratification (ESC/EAS 2019 algorithm).
  - LDL-C target determination.
  - Intensification pathway:
    ▪ Maximize statin (with safety monitoring).
    ▪ Add ezetimibe if target not achieved.
    ▪ Consider PCSK9i for very high-risk patients.
Triggers for Unscheduled In-Person Evaluation:
  - New/worsening chest pain or dyspnea at rest.
  - Syncope or presyncope.
  - Sustained BP >180/110 mmHg with symptoms.
  - Weight gain >3 kg in 5 days despite diuretic adjustment.
  - Patient/physician concern requiring physical examination.
Quality Assurance Measures:
  - 10% of consultations randomly recorded (with consent) for quality review.
  - Quarterly physician peer review sessions.
  - Patient satisfaction survey after each visit (5-item scale).
  - Technical quality metrics (connection failures and audio/video issues) tracked.
  - Consultation duration monitoring to ensure adequate time allocation.

**Sample size justification**

Sample size calculation is based on the primary composite endpoint of clinical control at 12 months. Based on pilot data and the published literature, we anticipate the following baseline control rates in standard care:

Hypertension: 35% achieving BP <130/80 mmHg.
Dyslipidemia: 40% achieving individualized LDL-C targets.
Heart failure: 50% achieving composite endpoint (no HF hospitalization + maintained/improved NYHA class).

We hypothesize that the AI-enhanced telemedicine intervention will improve clinical control rates by an absolute 20 percentage points (from approximately 40% to 60% across disease categories). Using a two-sided significance level of α = 0.05 and power of 80%, the required sample size is 97 patients per group. Accounting for an anticipated 15% dropout rate over 12 months, we will enroll 300 patients total (150 per group).

This sample size provides adequate power for primary endpoint analysis (chi-square test comparing proportions); key secondary analyses including continuous BP changes (paired t-tests), lipid parameter changes, and hospitalization rates (Poisson regression); and subgroup analyses by disease category (hypertension, dyslipidemia, and heart failure).

Statistical power calculations were performed using G Power 3.1 software. Interim analyses are not planned given the 12-month study duration and low risk profile of the intervention.

1. **Initial On-Site Consultation with AI-Assisted History Acquisition and Documentation**

All intervention patients commence with an in-person baseline visit where AI-based systems facilitate comprehensive data capture through the following:

*Pre-consultation AI-driven history collection:* Patients complete adaptive questionnaires eliciting cardiovascular symptoms, medical history, and home measurements. The AI system (based on natural language processing algorithms) synthesizes responses into structured preliminary documentation for physician review. The questionnaires are adaptive, with follow-up questions dynamically generated based on initial responses to ensure comprehensive data capture while minimizing patient burden.

*Physician validation:* Treating physicians review, edit, and validate AI-generated drafts for accuracy and clinical appropriateness. The finalized note enters both the institutional EHR and the patient’s application profile, establishing a comprehensive baseline telemedicine record. All AI-generated documentation requires mandatory physician approval before being finalized in the EHR.

2. **Telemonitoring and Virtual Follow-Up Consultations**

Following baseline assessment, patients transition to remote disease management for 12 months, incorporating the following:

*Regular home physiologic measurements:* Hypertensive patients perform protocol-specified blood pressure monitoring (twice daily for first week, then 3 times weekly, using validated oscillometric devices provided by the study); heart failure patients measure daily weight and blood pressure with structured symptom assessment (using a standardized questionnaire integrated into the mobile application); and dyslipidemia patients undergo scheduled laboratory testing at months 3, 6, 9, and 12, with results uploaded to the platform. Validated monitoring devices transmit data automatically via device–application pairing. All devices are calibrated and validated according to international standards (ESH/AAMI protocols for BP monitors).

*Continuous data integration and AI-driven summarization:* The AI analyzes patient-reported data and physiologic measurements in real-time, tracking trends and flagging clinically significant changes. Auto-generated summaries synthesize data streams for physician dashboard display (e.g., “Home blood pressure averaged 130/80 mmHg this week, with one elevated reading of 145/92 mmHg”). Laboratory results trigger automated interpretation summaries, mitigating information overload. The AI system employs rule-based algorithms combined with machine learning models trained on cardiovascular clinical data to identify clinically significant patterns and trends.

*Scheduled telemedicine consultations:* Patients complete follow-up visits at protocol-specified intervals (monthly for first 3 months, then every 2 months) via video/telephone. Pre-consultation, patients complete symptom questionnaires synthesized by AI for clinician review. During teleconsultations, physicians access AI-generated summaries; the AI assistant transcribes conversations and generates draft progress notes for physician approval. All teleconsultations are conducted via a HIPAA-compliant, encrypted video platform integrated with STmedical.

*Patient education, clinical alerts, and unscheduled care access:* Patients receive instruction on scenarios requiring urgent in-person evaluation (severe chest pain, marked dyspnea, syncope, and hypertensive crisis, defined as BP > 180/120 mmHg with symptoms). The system generates automated alerts for pre-defined thresholds: BP > 160/100 mmHg on 2 consecutive readings; weight gain >2 kg in 3 days (HF patients); and patient-reported severe symptoms (chest pain, dyspnea at rest, and syncope). Throughout the intervention, guideline-directed pharmacotherapy continues; the AI aggregates information without autonomous treatment decisions. Therapeutic changes are communicated through teleconsultations with appropriate follow-up scheduling. Patients have 24/7 access to an emergency contact number for urgent clinical concerns.

Quality assurance includes mandatory physician review of all AI-generated documentation. Inaccurate summaries trigger physician correction with formal error tracking to quantify potential AI-related documentation issues. A standardized error classification system will categorize documentation errors by type and severity.

3. **Automated Synthesis of Patient-Reported Data and Laboratory Results**

The AI continuously updates a longitudinal journal within STmedical, processing symptom diaries and laboratory results. AI modules compare values to prior results and guideline targets, updating physician dashboards with temporal trend analysis presented via chronological timeline visualization. Patients access simplified data summaries via mobile application with AI-generated educational insights in lay language, providing informational support within physician-approved parameters.

The AI system architecture includes a natural language processing module (for symptom questionnaire analysis); trend detection algorithms (for physiologic parameter monitoring); a clinical decision support module (providing guideline-based recommendations but not autonomous treatment decisions); and a documentation generation module (creating structured clinical notes from encounter data).The system undergoes continuous quality monitoring with performance metrics tracked throughout the study.

**Figure 3:**
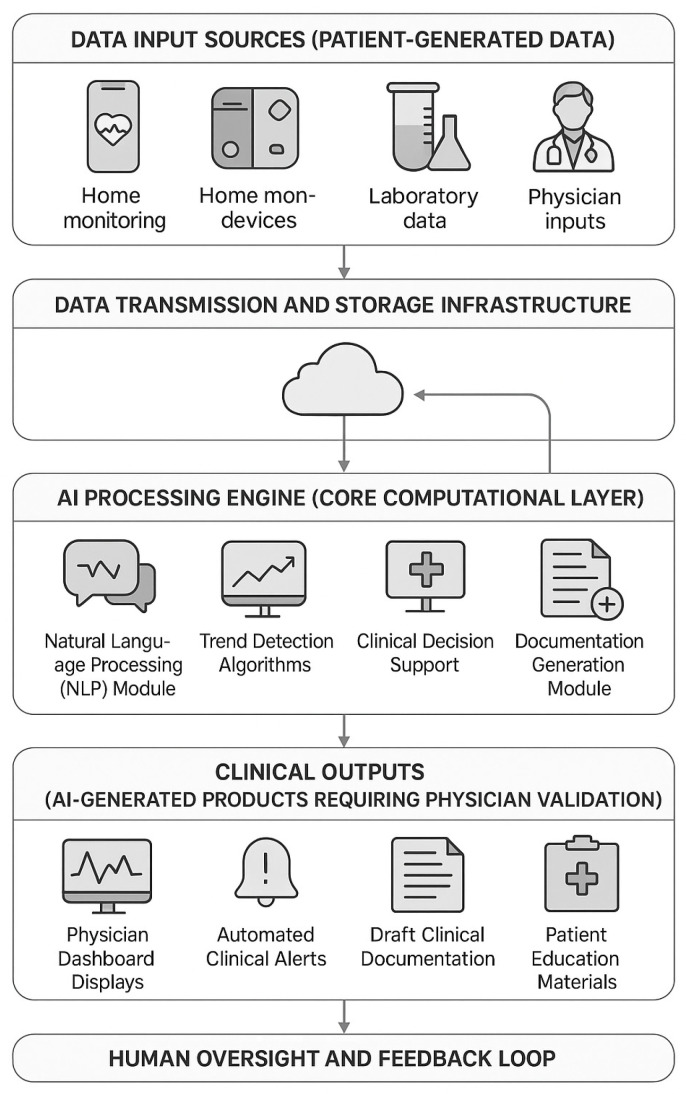
AI system architecture and data flow in TeleAI-CVD platform.

The intervention provides coordinated telemedicine delivery: initial in-person AI-supported evaluation, serial remote monitoring with AI-driven summarization, and on-demand care access, all superimposed upon guideline-directed pharmacotherapy. This technology-enhanced approach facilitates responsive clinical management (earlier medication adjustments, prompt symptom-based interventions, and reinforced counseling) with AI functioning as workflow optimization rather than a clinical decision-making replacement (Figure 3).

### 6.5. Control Group: Standard Ambulatory Care

Control patients receive usual outpatient cardiology care without telemedicine or AI assistance, reflecting current standard practice: comprehensive baseline cardiologist evaluation with manual EHR documentation, physical examination, and indicated diagnostic testing, concluding with patient education and follow-up planning.

In-person appointments are per standard practice (typically 6–12 month intervals for stable hypertension/dyslipidemia and 3–6 months for heart failure, adjusted by clinical judgment). No routine telephone contact or telemedicine check-ins occur; interim contact is patient-initiated.

Follow-up encounters assess symptoms, vital signs, and adherence, with appropriate therapeutic adjustments. Clinical decisions rely on intermittently available data (office measurements and patient-reported logs). Protocol-specified laboratory testing matches intervention timing (months 3, 6, 9, and 12) for valid outcome comparison. No AI documentation assistance or automated trend analysis is available; physicians access only patient-provided data at scheduled visits.

Control patients receive comparable disease management education; the primary differential lies in delivery modality and monitoring intensity (continuous remote monitoring versus periodic in-person assessment). This design isolates the specific impact of telemedicine plus AI integration.

### 6.6. Outcomes

The primary outcome of this study is the assessment of clinical control at 12 months using a composite endpoint: for hypertension, BP control (<130/80 mmHg based on an average of 3 home readings over the final week); for dyslipidemia, target LDL-C attainment (individualized based on ESC/EAS cardiovascular risk category); for heart failure, composite of no HF hospitalizations during study period and maintained or improved NYHA class at 12 months. “Successful clinical control” requires that all applicable targets are met for each patient’s specific diagnoses. For patients with multiple conditions, all applicable criteria must be satisfied. Primary analysis compares 12-month proportions between groups, with continuous parameter changes as supportive analyses.

A comprehensive range of secondary endpoints will be assessed to capture the multifaceted impact of the intervention:

**Patient Satisfaction and Engagement:** Validated questionnaires at 6 and 12 months comparing satisfaction scores and qualitative feedback.

**Physician and Healthcare Provider Experience:** Post-study surveys evaluating documentation burden (time per patient encounter and after-hours EHR work); validated burnout assessment (Maslach Burnout Inventory—Human Services Survey); work–life balance evaluation; hypothesized reduced charting time and EHR frustration.

**Clinical Efficacy Outcomes:** BP changes (mean systolic and diastolic BP at 6 and 12 months compared to baseline); lipid improvements (LDL-C, HDL-C, triglycerides, and non-HDL-C at 6 and 12 months); HF hospitalization incidence (number and duration); all-cause hospitalization rates (days per patient-year); all-cause mortality (descriptive analysis, study not powered for mortality); NYHA class changes (proportion with improvement, stability, and deterioration); quality-of-life scores (Kansas City Cardiomyopathy Questionnaire for HF patients, EQ-5D-5L for all patients); medication adherence (Morisky Medication Adherence Scale-8); time to therapeutic target achievement.

**Safety Outcomes:** Systematic documentation of medical- and technology-related adverse events (symptomatic hypotension, syncope, electrolyte abnormalities, adverse drug reactions, delayed care due to technical failures, and misinterpretation of telemonitoring data); AI documentation errors (categorized by type: factual errors, omissions, and misinterpretations); telemonitoring failures (device malfunctions and data transmission failures); mandatory tracking protocols with standardized adverse event reporting forms; independent safety monitoring committee reviewing serious adverse events.

**Health Economic and Utilization Outcomes:** Total clinic visits (in-person and virtual); emergency department visits; hospitalizations (cardiovascular and all-cause, days and costs); diagnostic testing utilization; cost-effectiveness analysis if benefit demonstrated; incremental cost per patient achieving targets; cost per hospitalization prevented; cost per quality-adjusted life year (QALY) gained; physician time analysis (time per patient encounter: documentation, review, decision-making, and after-hours EHR work); qualitative assessment of workflow efficiency.

All outcomes will be operationally defined a priori in a detailed statistical analysis plan, which will be finalized and registered before database lock. Primary and key secondary endpoints will be evaluated using pre-specified statistical tests to comprehensively assess AI-integrated telemedicine efficacy.

### 6.7. Data Collection Procedures

Baseline data: Demographic characteristics, medical history, medications, laboratory parameters, vital signs, and disease-specific assessments (NYHA class and cardiovascular risk stratification).

Intervention group: Automated collection via telemonitoring platform (BP, weight, and symptom questionnaires), EHR extraction (medications, laboratory results, and clinical encounters), and patient-reported outcome measures at 6 and 12 months.

Control group: EHR extraction at protocol-specified timepoint and patient-reported outcome measures at 6 and 12 months.

Endpoint adjudication: Independent review of hospitalizations and major adverse events using standardized criteria.

#### 6.7.1. Data Management

Electronic case report forms: eCRFs in REDCap secure database.

Data validation: Automated range checks, logic checks, and manual review by study coordinators.

Data monitoring: Regular quality checks and site monitoring visits (virtual and in-person).

Data security: HIPAA-compliant encrypted storage, role-based access controls, and audit trails.

Missing data handling: Multiple imputation for missing outcome data in primary analyses; complete case analysis as sensitivity analysis.

#### 6.7.2. Data Quality Assurance

Training of all study personnel on data collection protocols.

Standard operating procedures for all measurements and assessments.

Regular calibration of devices (BP monitors and weight scales).

Independent data verification for 10% of randomly selected records.

#### 6.7.3. Statistical Analysis Plan

Primary analysis: Intention-to-treat principle (all randomized patients analyzed according to assigned group regardless of adherence); significance level α = 0.05 (two-sided); effect estimates (risk ratio and risk difference with 95% confidence intervals).

Secondary analyses: Continuous outcomes (BP, lipids, quality-of-life scores, linear mixed-effects models with repeated measures, adjusted for baseline values, stratification variables, and relevant covariates); time-to-event outcomes (hospitalizations—Kaplan–Meier curves and Cox proportional hazards models); count outcomes (number of hospitalizations and clinic visits—negative binomial regression); categorical outcomes (NYHA class changes—ordinal logistic regression).

Subgroup analyses (exploratory): By disease category (hypertension, dyslipidemia, and heart failure); by age (<65 vs. ≥65 years); by baseline disease severity; by baseline technology literacy; interaction tests (will assess whether treatment effects differ across subgroups).

Sensitivity analyses: Per-protocol analysis (excluding patients with major protocol deviations); complete case analysis (excluding patients with missing outcome data); tipping point analysis (assessing robustness to missing data assumptions).

Handling of multiple comparisons: Primary endpoint tested at α = 0.05; key secondary endpoints (BP change, lipid change, and HF hospitalizations—tested using Hochberg procedure to control family-wise error rate); other secondary endpoints reported with nominal *p*-values and confidence intervals (interpreted as exploratory).

Interim analyses: No formal interim efficacy analyses planned.

Safety monitoring: Ongoing review by independent safety monitoring committee; study may be stopped for safety concerns.

Statistical software: R version 4.0 or later and SAS version 9.4 or later.

A detailed, comprehensive statistical analysis plan will be finalized and registered on ClinicalTrials.gov before database lock and will specify all analytical methods, handling of missing data, sensitivity analyses, and subgroup analyses in full detail.

### 6.8. Feasibility and Implementation

This protocol represents a fully implementable study design based on the following:

1. Established infrastructure: Existing outpatient cardiology clinics with electronic health record systems, trained clinical staff, and patient population.
2. Validated technology platform: STmedical telemedicine platform with proven functionality in clinical settings and an integrated AI documentation system with preliminary validation.
3. Regulatory approvals: Ethics committee approval obtained (IRB/ERC of the Kosice Self-Governing Region, Reference number 9483/2025/ODDZ-48993).
4. Funding secured: Grant VEGA 1/0700/23 provides financial support for study conduct.
5. Operational readiness: Standard operating procedures developed, staff training completed, and device procurement arranged.

### 6.9. Timeline

Enrollment: 12 months (25 patients/month).

Follow-up: 12 months per patient.

Data analysis and reporting: 6 months post-enrollment completion.

Total study duration: approximately 30 months.

Quality assurance mechanisms: Regular investigator meetings; protocol adherence monitoring; technology performance monitoring; independent data and safety monitoring.

## 7. Results and Discussion

This study distinguishes itself from prior investigations by endeavoring to comprehensively evaluate the complete spectrum of clinical data and parameters for each enrolled patient, encompassing rigorously analyzed and accurately interpreted anamnestic information and diagnostic modalities

We anticipate the following outcomes from this study:

Primary hypothesis: The AI-enhanced telemedicine intervention will significantly increase the proportion of patients achieving clinical control at 12 months compared to standard care, with an absolute improvement of approximately 20 percentage points (from 40% to 60%).

Secondary hypotheses:

1. Clinical efficacy: Greater reductions in BP (estimated 5–10 mmHg additional systolic BP reduction), larger improvements in lipid parameters (estimated 15–20 mg/dL additional LDL-C reduction), and reduced HF hospitalization rates (estimated 30–40% relative reduction).
2. Patient experience: Higher satisfaction scores, improved quality of life, and better medication adherence in the intervention group.
3. Physician experience: Reduced documentation burden (estimated 20–30% reduction in time per patient), decreased burnout scores, and improved work–life balance despite increased patient monitoring intensity.
4. Safety profile: Comparable or improved safety outcomes with no increase in adverse events, with potential for earlier detection and intervention for clinical deterioration.
5. Healthcare utilization: Reduced total healthcare utilization despite increased virtual encounters, with particular reductions in emergency department visits and hospitalizations.

Our objective is to facilitate the timely and appropriate utilization of individual diagnostic methods while simultaneously obviating their unnecessary deployment in circumstances where clinical indication is absent. We posit that through the synergistic integration of precision medicine principles with machine learning algorithms, we may contribute meaningfully to the amelioration of cardiovascular morbidity and mortality in the ambulatory care setting. Each patient represents a unique biological entity and accordingly merits an individualized therapeutic approach. Without leveraging contemporary computational technologies and data analytics, such personalized care would likely remain an unattainable ideal.

This project will elucidate previously unexplored interconnections and etiological significance among individual cardiovascular risk factors across multiple hierarchical levels—relationships that have historically been investigated in isolation due to inherent methodological constraints of conventional research paradigms and the underutilization of advanced artificial intelligence capabilities in the analysis of large-scale ambulatory cardiology databases.

Our central hypothesis posits that substantial overutilization of various diagnostic procedures and imaging modalities can be mitigated through more expansive implementation of precision medicine frameworks integrated with artificial intelligence methodologies. We anticipate that this investigation will engender a novel clinical decision support system predicated upon precision medicine principles and leveraging the totality of available patient-specific data, thereby optimizing diagnostic accuracy while minimizing healthcare resource expenditure and patient exposure to unnecessary interventions.

Potential limitations and mitigation strategies: Open-label design (mitigated through objective outcomes, blinded adjudication, and blinded statistical analysis); technology adoption challenges (mitigated through careful patient selection, comprehensive training, and ongoing technical support); single-center design (limits generalizability but enables rigorous protocol adherence and quality control); AI algorithm performance (continuous monitoring and quality assurance with human oversight of all AI-generated content).

If successful, this study will provide the following:

1. Evidence base—for AI-enhanced telemedicine in cardiovascular care.
2. Operational framework—for implementation in other healthcare settings.
3. Health economic data—to inform healthcare policy and reimbursement decisions.
4. Foundation—for future multi-center trials and broader implementation.

## Figures and Tables

**Table 1:** Estimated patient characteristics and target distribution (*N* = 300).

Characteristic	Target Distribution	Rationale
Total sample size	300 patients (150 per group)	Powered for 20% absolute difference in primary endpoint
Disease distribution		Stratified randomization
- Hypertension only	35% (*n* = 105)	Largest ambulatory cardiology population
- Dyslipidemia only	25% (*n* = 75)	Common isolated condition
- Heart failure only	20% (*n* = 60)	High-risk, resource-intensive
- Multiple conditions	20% (*n* = 60)	Reflects real-world comorbidity
**Age distribution**		**Representative of ambulatory cardiology**
- <65 years	40% (*n* = 120)	Working-age population
- ≥65 years	60% (*n* = 180)	Higher CVD prevalence
**Sex distribution**		**Target equal representation**
- Male	50% (*n* = 150)	
- Female	50% (*n* = 150)	
**Technology literacy**		**Inclusion criterion**
- Comfortable with devices	70% (*n* = 210)	Facilitates adoption
- Requires caregiver support	30% (*n* = 90)	Maintains equity
**Baseline control status**		**Enrichment for intervention benefit**
- Suboptimal control	70% (*n* = 210)	Primary target population
- At-target but high-risk	30% (*n* = 90)	Prevention focus

## Data Availability

The datasets used and/or analyzed during the current study are available from the corresponding author on reasonable request due to legal reasons.
